# Supplementary material for: Causal Relationship between Aging and Anorexia Nervosa: A White-Matter-Microstructure-Mediated Mendelian Randomization Analysis
Source: Biomedicines. 2024 Aug 16;12(8):1874. doi: 10.3390/biomedicines12081874 (PMC11351342; doi:10.3390/biomedicines12081874)
Supplement: Supplementary file 1 [file biomedicines-12-01874-s001.zip › Supplementary Figure/Figure S8/Leaveoneout plot of MR analysis for TL to ubm-b-2065.pdf]

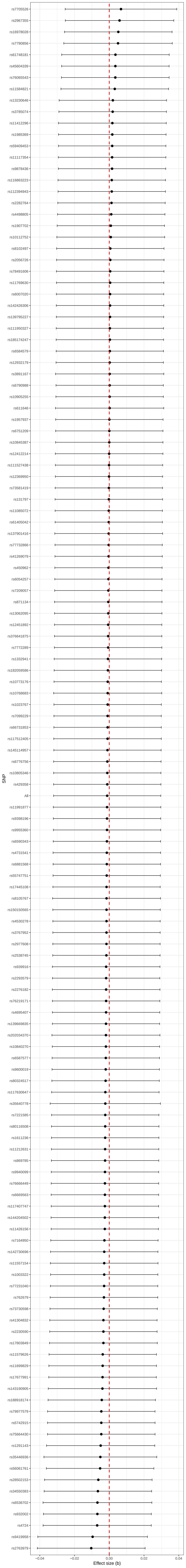

rs7705526
rs2967355
rs16978028
rs7790856
rs61748181
rs45604339
rs76065543
rs11584821
rs13230646
rs3785074
rs11412296
rs1985369
rs59409453
rs11117354
rs9878436
rs116863223
rs112394943
rs2282764
rs4498805
rs1907702
rs10112752
rs8102497
rs2056726
rs78491606
rs11769630
rs6007020
rs142426306
rs139795227
rs111950327
rs185174247
rs6584579
rs12932179
rs3891167
rs6790988
rs10905255
rs611646
rs1957937
rs6751209
rs10845387
rs12412214
rs111527438
rs12369950
rs73581419
rs131797
rs11085072
rs61405042
rs137901416
rs77732866
rs41269079
rs450962
rs6054257
rs7209057
rs871134
rs13062095
rs12451892
rs376641875
rs7772289
rs1332941
rs182059586
rs10773176
rs10768683
rs1023767
rs7099229
rs66731853
rs117512405
rs145114957
rs6776756
rs10805346
rs429358
A8
rs11991877
rs9398196
rs9955360
rs6190343
rs4731541
rs6881568
rs55747751
rs17445108
rs8105767
rs150150565
rs4530278
rs3767952
rs2977608
rs2538745
rs939916
rs2293579
rs2276182
rs76219171
rs4695407
rs139669835
rs202034370
rs10840270
rs6587577
rs9600019
rs80324517
rs117630647
rs35640778
rs7221585
rs80116508
rs1611236
rs11212631
rs869785
rs9940099
rs76666449
rs6669563
rs117407747
rs144204502
rs11426156
rs7164950
rs142730696
rs11557154
rs1003322
rs77231040
rs762679
rs73730598
rs41304832
rs2230590
rs17803849
rs11579626
rs11699829
rs17677991
rs143190905
rs188918174
rs79977579
rs5742915
rs75664430
rs1291143
rs35446936
rs56061761
rs28502153
rs34550383
rs6536702
rs932002
rs4724
rs9419958
rs2763979
SNP
-0.04
-0.02
0.00
0.02
0.04
Effect size (b)
